# Exploring the Pregnant Guinea Pig as a Model for Group B Streptococcus Intrauterine Infection

**DOI:** 10.4172/2576-1420.1000109

**Published:** 2017-07-27

**Authors:** Maria I Harrell, Kellie Burnside, Christopher Whidbey, Jay Vornhagen, Kristina M Adams Waldorf, Lakshmi Rajagopal

**Affiliations:** 1 Department of Pediatrics, University of Washington and Seattle Children’s Research Institute, Seattle, Washington, United States of America; 2 Department of Global Health, University of Washington, Seattle, Washington, United States of America; 3 Department of Obstetrics and Gynecology, School of Medicine, University of Washington, Seattle, Washington, United States of America

**Keywords:** Pregnant guinea pig, Streptococcus, Intrauterine, Infection

## Abstract

Infection of the amniotic cavity remains a major cause of preterm birth, stillbirth, fetal injury and early onset, fulminant infections in newborns. Currently, there are no effective therapies to prevent in utero infection and consequent co-morbidities. This is in part due to the lack of feasible and appropriate animal models to understand mechanisms that lead to *in utero* infections. Use of mouse and rat models do not fully recapitulate human pregnancy, while pregnant nonhuman primate models are limited by ethical considerations, technical constraints, and cost. Given these limitations, the guinea pig is an attractive animal model for studying pregnancy infections, particularly as the placental structure is quite similar to the human placenta. Here, we describe our studies that explored the pregnant guinea pig as a model to study *in utero* Group B Streptococci (GBS) infections. We observed that intrauterine inoculation of wild type GBS in pregnant guinea pigs resulted in bacterial invasion and dissemination to the placenta, amniotic fluid and fetal organs. Also, hyperhemolytic GBS such as those lacking the hemolysin repressor CovR/S showed increased dissemination into the amniotic fluid and fetal organs such as the fetal lung and brain. These results are similar to those observed in mouse and non-human primate models of *in utero* infection, and support use of the guinea pig as a model for studying GBS infections in pregnancy.

## Introduction

Group B Streptococci are β-hemolytic gram-positive bacteria that commonly reside in the lower gastrointestinal tract of healthy women. However, an ascending infection of GBS from the vagina into the uterus during pregnancy increases the risk of preterm birth, stillbirth and early onset newborn infections. Despite observations that link GBS colonization of the lower genital tract to chorioamnionitis, rupture of membranes and transmission to the fetus, the mechanisms that promote ascending infection are not completely understood [1–3]. How changes in host pathogen interactions influence ascending infection are not well described [3]. Additionally, environmental factors that promote ascending infection of GBS are not completely elucidated. Furthermore, the lack of appropriate animal models contributes to the knowledge gap on infections that occur during pregnancy. This is because no animal model fully recapitulates human pregnancy. Also, single species animal models are limited in their ability to fully reproduce human physiology. In order to successfully replicate human disease, especially those that occur during complex physiological processes such as pregnancy, multiple experimental models are necessary. Recently, much work has been done to develop the pregnant mouse and non-human primate models of GBS infection during pregnancy [4–11]. Although pregnancy in nonhuman primates more closely resembles human pregnancy, their application is limited due to constraints on ethical use, requirement of a large team of specialized experts and costs [9–12]. While the mouse is commonly used to study infections during pregnancy, limitations include key differences with human pregnancy in the mechanism of parturition, uterine and placental structure, gestational length and sensitivity to common perinatal pathogens [9].

The pregnant guinea pig (*Cavia porcellus*) is a closer model of human pregnancy based on similarities in progesterone levels across gestation and at the time of parturition, placental structure (i.e., hemomonochorial), deep trophoblast invasion and remodelling of the maternal spiral arteries, sensitivity to pathogens, prolonged gestation (~67 days in guinea pig versus ~21 days in mouse) and advanced maturity of the neonate [13–18]. Pregnant guinea pigs are considered to be a highly relevant non-primate animal model for studies of chronic placental hypoxia [18], fetal growth restriction and multiple bacterial (e.g. *L. monocytogenes, E. coli* and *C. trachomatis*) and viral infections [19–25] (e.g. Zika virus [26], cytomegalovirus [27]). The addition of the guinea pig model to the mouse and non-human primate models will increase the relevance of factors that enable GBS establish infections during pregnancy.

**Figure 1:**
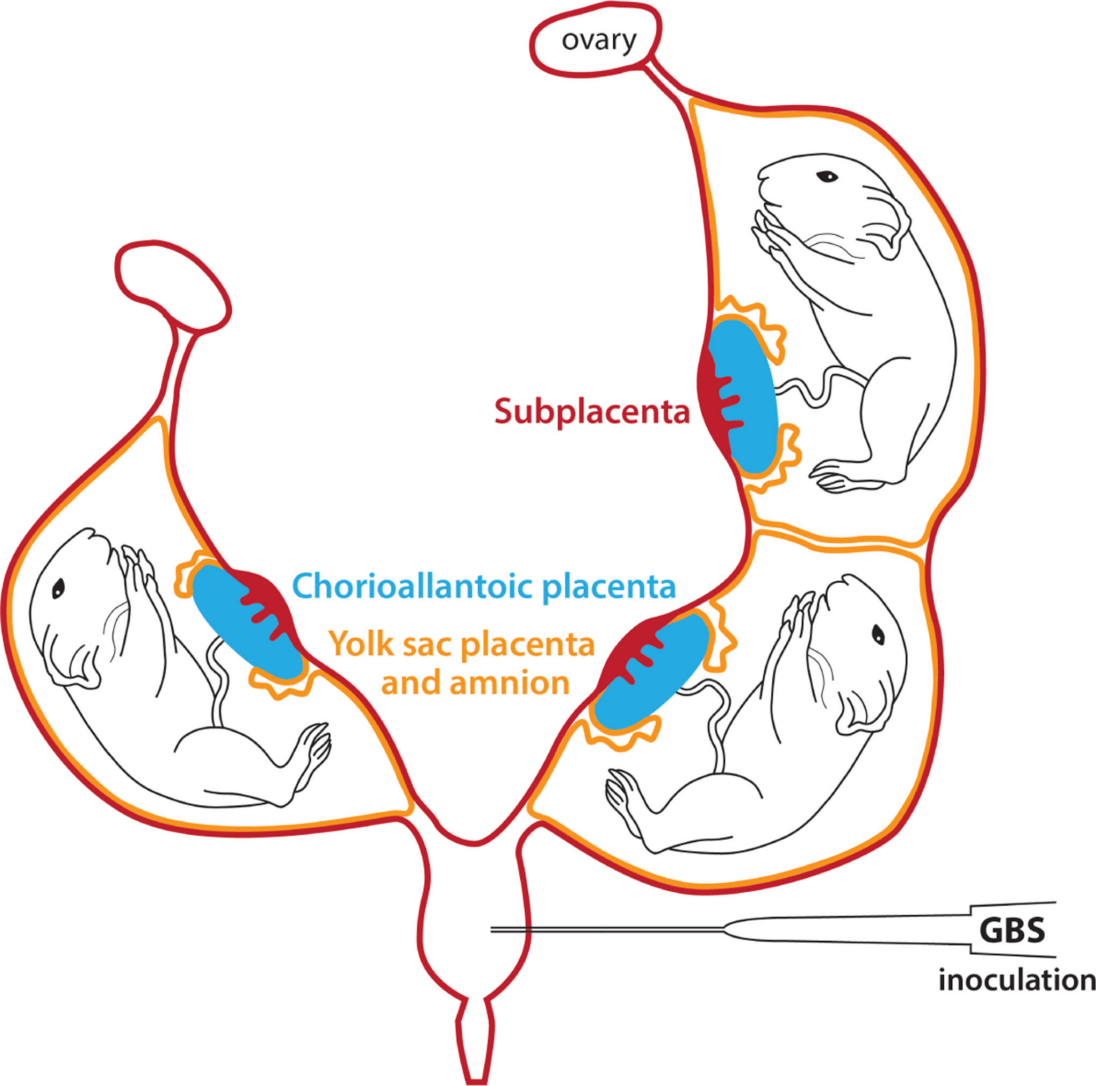
Schematic representation of the bicornuate guinea pig uterus with fetal sacs in each horn.

To confirm that the pregnant guinea pig would be useful for studies on GBS infection during pregnancy, we adapted an established mouse model of intrauterine infection [28–30] to the guinea pig. Pregnant Hartley guinea pigs at ~39–40 days gestation (term ~59–72 days) were obtained from Elm Hill Labs, MA, USA. To expose the uterine horns, a midline laparotomy was performed on pregnant guinea pigs at 45 days gestation under isoflurane anaesthesia, as described for the mouse model [28–30]. The guinea pig uterus is bicornuate and may contain between 1–6 fetuses during pregnancy. Two membranes known as the yolk sac placenta and amnion enclose each fetus (Figure 1). The yolk sac placenta is the anatomical and functional equivalent of the human chorion, and the guinea pig amnion has structural similarity to the human amnion [16–31,32]. As cervical or vaginal inoculations may result in inconsistent pregnancy outcomes, GBS was inoculated directly into the uterus inferior to the lowest pup to allow bacterial spread into both uterine horns. Either saline (*n*=1) or 10^7^ colony forming units (CFU) of a wild-type (WT) GBS strain (serotype III, COH-1; *n*=2) or an isogenic hyper-virulent, and hyper-hemolytic GBS strain (GBSΔcovR, *n*=2) was injected between the uterine horns, with care not enter a fetal sac or placenta (Figure 1). The uterus was then returned to the abdomen, which was then closed using absorbable suture. The animals recovered within 10 min of the procedure and were observed for signs of distress or morbidity (piloerection, vaginal bleeding, and preterm delivery). To confirm GBS dissemination into fetal tissues, we terminated the experiment at ~8 hrs post-infection when the animals did not exhibit signs of distress or evidence of preterm labor. Guinea pigs were euthanized by humane means and a necropsy was performed. Amniotic fluid, and fetal organs, such as the lung and brain were harvested from each fetus individually. Bacterial CFU were enumerated in various tissues.

**Figure 2:**
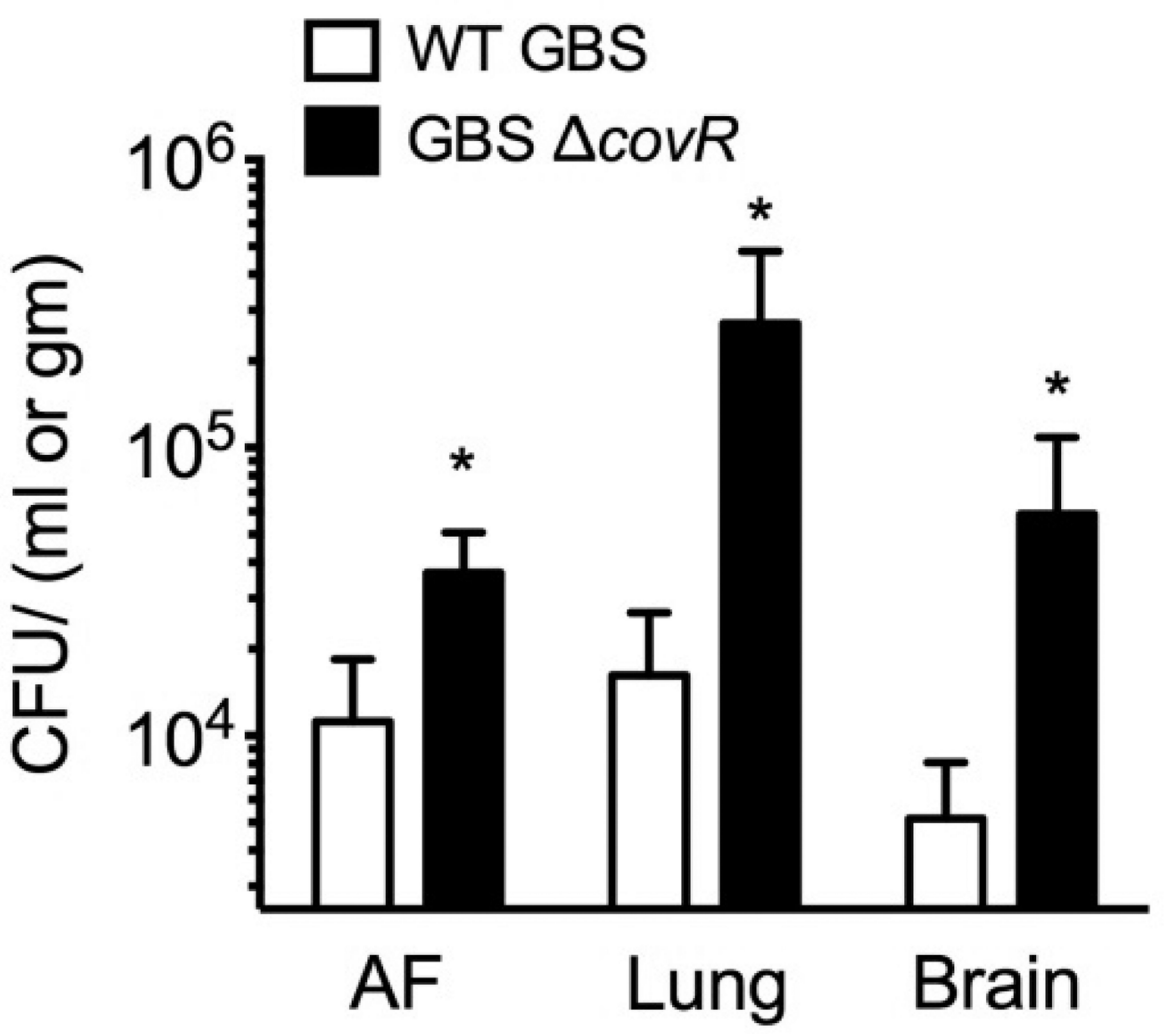
Intrauterine inoculation of GBS in pregnant guinea pigs results in bacterial invasion of amniotic fluid (AF), fetal lung and fetal brain.

The results shown in Figure 2 indicate that WT GBS are able to invade placental membranes, survive in amniotic fluid and penetrate fetal organs such as the lung, and brain in the pregnant guinea pig model. The hypervirulent GBS strain (GBSΔcovR lacking the hemolysin repressor CovR/S) used in this study has previously been associated with increased placental invasion and preterm births [7,30,33]. Consistent with these observations, GBSΔcovR exhibited increased invasion/dissemination when compared to the WT strain (Figure 2). Of note, we did not recover bacteria from the saline control animal.

Our studies are the first to demonstrate that intrauterine inoculation outside the fetal sacs results in GBS invasion of fetal lung and brain in the pregnant guinea pig model. Further studies are needed for evaluation of the guinea pig as an appropriate model for studies of GBS infection-associated preterm birth and stillbirth.

## Discussion

Although the pregnant guinea pig is more similar to human when compared to murine models, disadvantages of the guinea pig include greater cost, genetic intractability and limited reagents (e.g. guinea pig-specific antibodies) when compared to mice; however, new guinea pig-specific reagents are regularly being developed [24]. Nevertheless, our initial observations with the pregnant guinea pig links intrauterine inoculation of GBS to bacterial invasion of placenta, amniotic fluid and fetal organs, which mirror what is observed in the pregnant mouse model and the nonhuman primate model of *in utero* GBS infection [7,30]. These results confirm the feasibility of this animal model for additional studies of GBS infections.

While intrapartum prophylactic antibiotic administration during labor and delivery has proven to be effective in reducing the burden of neonatal GBS disease, it has not halted disease incidence. Additionally, ascending GBS infection is highly associated with stillbirth and preterm birth. A significant portion of these infections are attributable to *in utero* infections early in pregnancy, yet little is known about how these infections occur. Multiple studies have described the role of the GBS hemolytic pigment in ascending and *in utero* infection [5,7,30], and our data here corroborate these findings in the pregnant guinea pig model. Moreover, GBS that overexpress the hemolytic pigment have been isolated from women undergoing preterm labor and in other cases of severe infection [33–36]. Establishment of the role of a virulence factor in bacterial disease pathogenesis is necessary for identification and testing of therapeutics and vaccines. Prevention of infections during pregnancy requires testing in many animal models prior to clinical trials due to differences in aspects of pregnancy in the various animal models and sensitivity of the developing fetus. Our studies show that pregnant guinea pig can serve as an appropriate model for studies on GBS infections during pregnancy.

All animal experiments were approved by the Seattle Children’s Research Institutional Animal Care and Use Committee (protocol #13907) and performed in strict accordance with the recommendations in the Guide for the Care and Use of Laboratory Animals of the National Institutes of Health (8^th^ Edition). All surgery was performed with appropriate anaesthesia and analgesia, and every effort was made to minimize suffering.

Pregnant guinea pigs at ~39–40 days gestation (term 59–72 days) were obtained from Elm Hill Labs, MA, USA. Briefly, on 45 days gestation, dams were anesthetized using isoflurane (3–4%) via nose cone and maintained under anaesthesia during the duration of the procedure. Sterile surgical techniques were used. Subcutaneous injection of buprenorphine (0.025 mg/kg, (Webster Veterinary Supply) was administered pre-operatively.

The surgical site at the caudal abdomen was shaved and prepared using triple alternation of betadine scrub /alcohol scrub around surgical site. Subsequently, a ventral midline laparotomy was performed to expose uterine horns via midline incisions into the caudal abdominal skin and peritoneum as described for mice [29]. Approximately, 10^7^ CFU (100 µL) of GBS was injected between the uterine horns inferior to the lowest pup. After inoculation, sterile saline was applied to the exposed uterus, and the uterus was returned to the abdomen. Subsequent to GBS inoculation, the abdomen was closed in 3 layers (muscle, subcutaneous using absorbable suture (Vicryl 3-0, Medline Industries) and intradermal and/or skin with absorbable suture and skin glue as needed (Dermabond skin adhesive, Medline industries). Replacement fluids (10 ml/kg/hr sterile 0.9% NaCl or lactated ringers solution) were given subcutaneously at the conclusion of surgery. The animals were placed in a recovery cage on a heating pad and observed until ambulatory and then every 2 hrs post-surgery till experimental end at 8 hrs, at which point they did not exhibit preterm birth (vaginal bleeding and pup in cage) or morbidity symptoms (ruffling of fur, not eating/drinking, lack of spontaneous movement, fatigue, labored breathing, lethargy or significant weight loss, >10%). Animals were euthanized at 8 hours post infection using anesthesia first with isoflurane (5% in an induction chamber) and then, following no response to toe pinch, 100 mg/kg of pentobarbital (Webster Veterinary Supply) was injected intraperitoneally or intracardiac. Fetuses were euthanized by decapitation and fetal tissues were collected and homogenized for enumeration of bacterial CFU by serial dilution and plating using methods described [7].
